# Microneedle Array-Assisted, Direct Delivery of Genome-Editing Proteins Into Plant Tissue

**DOI:** 10.3389/fpls.2022.878059

**Published:** 2022-06-24

**Authors:** Anchu Viswan, Ayana Yamagishi, Masamichi Hoshi, Yuichi Furuhata, Yoshio Kato, Natsumi Makimoto, Toshihiro Takeshita, Takeshi Kobayashi, Futoshi Iwata, Mitsuhiro Kimura, Takeshi Yoshizumi, Chikashi Nakamura

**Affiliations:** ^1^Department of Biotechnology and Life Science, Tokyo University of Agriculture and Technology, Tokyo, Japan; ^2^Cellular and Molecular Biotechnology Research Institute, National Institute of Advanced Industrial Science and Technology (AIST), Tsukuba, Japan; ^3^Biomedical Research Institute, National Institute of Advanced Industrial Science and Technology (AIST), Tsukuba, Japan; ^4^Sensing System Research Center, National Institute of Advanced Industrial Science and Technology (AIST), Tsukuba, Japan; ^5^Graduate School of Medical Photonics, Shizuoka University, Hamamatsu, Japan; ^6^Faculty of Agriculture, Takasaki University of Health and Welfare, Takasaki, Japan

**Keywords:** genome editing, Cas9, Cre recombinase, *Arabidopsis* leaf, soybean SAM, microneedle array (MNA), direct delivery

## Abstract

Genome editing in plants employing recombinant DNA often results in the incorporation of foreign DNA into the host genome. The direct delivery of genome-editing proteins into plant tissues is desired to prevent undesirable genetic alterations. However, in most currently available methods, the point of entry of the genome-editing proteins cannot be controlled and time-consuming processes are required to select the successfully transferred samples. To overcome these limitations, we considered a novel microneedle array (MNA)-based delivery system, in which the needles are horizontally aligned from the substrate surface, giving it a comb-like configuration. We aimed to deliver genome-editing proteins directly into the inner layers of leaf tissues; palisade, the spongy and subepidermal L2 layers of the shoot apical meristem (SAM) which include cells that can differentiate into germlines. The array with needles 2 μm wide and 60 μm long was effective in inserting into *Arabidopsis thaliana* leaves and *Glycine max* (L.) Merr. (soybeans) SAM without the needles buckling or breaking. The setup was initially tested for the delivery of Cre recombinase into the leaves of the reporter plant *A. thaliana* by quantifying the GUS (β-glucuronidase) expression that occurred by the recombination of the *loxP* sites. We observed GUS expression at every insertion. Additionally, direct delivery of Cas9 ribonucleoprotein (RNP) targeting the PDS11/18 gene in soybean SAM showed an 11 bp deletion in the Cas9 RNP target site. Therefore, this method effectively delivered genome-editing proteins into plant tissues with precise control over the point of entry.

## Introduction

Genome-editing by the CRISPR/Cas9 system in plants is an increasingly researched area for improving crops (Jaganathan et al., 2018). DNA vectors have been commonly used for the expression of Cas9 and sgRNA in plants using standard transformation methods, such as using *Agrobacterium tumifaciens*, polyethylene glycol (PEG)-mediated DNA uptake, and particle bombardment (Bortesi and Fischer, 2015). However, there is concern regarding unwanted modification of the host genome by integration of transferred exogenous DNA. To remove the incorporated DNA from the target genome, complicated steps, such as self-pollination or crossing, are necessary. Moreover, the constitutive expression of Cas9 and sgRNA from DNA vectors can cause off-target effects. Additionally, the optimum promoter for gene expression in target plant tissues is required for DNA-based genome editing. These issues can be avoided by the direct delivery of Cas9 ribonucleoprotein (RNP) into plants. This method does not use DNA, including promoter sequences, and can avoid the overexpression of Cas9 and associated off-target effects (Metje-Sprink et al., 2018). Thus, direct delivery of RNP is expected to be a safe method for developing genome-edited plants.

The multi-layered rigid plant cell wall is a major barrier for the efficient direct delivery of RNP into plant tissues. To avoid the cell wall, direct delivery of RNPs has mainly targeted the protoplast or embryo, either by PEG-mediated uptake (Woo et al., 2015; Andersson et al., 2018) or biolistics (Svitashev et al., 2016; Liang et al., 2017, 2018). However, tissue cultures for regeneration of plants from protoplasts require a long time. Additionally, this method lacks versatility because it is only applicable for plant species that can be acceptable for regeneration from protoplasts, which is still a challenge for many plant species. To overcome the disadvantages of the protoplast-using method, which requires an *in vitro* cultivation step, the direct delivery of genome-editing protein into the shoot apical meristem (SAM), which has stem cells that continuously generate organs and tissues, has attracted attention (Murray et al., 2012). Out of the three layers in the SAM (L1, L2, and L3), the sub-epidermal L2 layer contains cells that develop into germ cells (Goldberg et al., 1993). If the cells in the L2 layer are genome-edited, germ cells differentiated from genome-edited cells can develop into seeds containing the edited genome. By cultivating plants from these seeds, genome-edited plants can be obtained without a regeneration step. Particle bombardment was reported for delivering Cas9 RNP into embryonic SAM of wheat and successfully obtained T_1_ individuals in which the target gene was knocked out (Imai et al., 2020). However, genome editing of SAM by direct delivery of RNP using particle bombardment has not been reported in other plant species. This may be due to the low efficiency of RNP delivery to the L2 layer since it is difficult to precisely control the position of the striking point by particle bombardment. In addition, particle bombardment can damage target cells because the microparticle introduced into the organelle could trigger stress for target cells. Although the mechanism has not been elucidated, the viability of the target cells by particle bombardment has been reported to be low (Lacroix and Citovsky, 2020).

Previously, we developed a nanoneedle technology for mammalian cells (Matsumoto et al., 2015). Nanoneedles 200 nm in diameter and 20 μm in length can be efficiently inserted into the cell membrane (Obataya et al., 2005) and we succeeded in efficient genome editing by direct delivery of Cas9 RNP using a nanoneedle array (Yamagishi et al., 2019). In this study, we use needle technology for plant manipulation. We aim to deliver genome-editing proteins directly into the inner layers of leaf tissues and subepidermal L2 layers of the SAM with a novel microneedle array (MNA) specific for the targets by optimizing various parameters such as needle length, width, and spacing between the needles. *Arabidopsis thaliana* as a model plant and soybean as a practical plant are used for direct delivery of Cre recombinase and Cas9 RNP, respectively. To the best of our knowledge, this is the first report of delivering genome-editing proteins into plant tissues with the help of an MNA.

## Materials and Methods

### Fabrication of Microneedle Array

**FIGURE 1:**
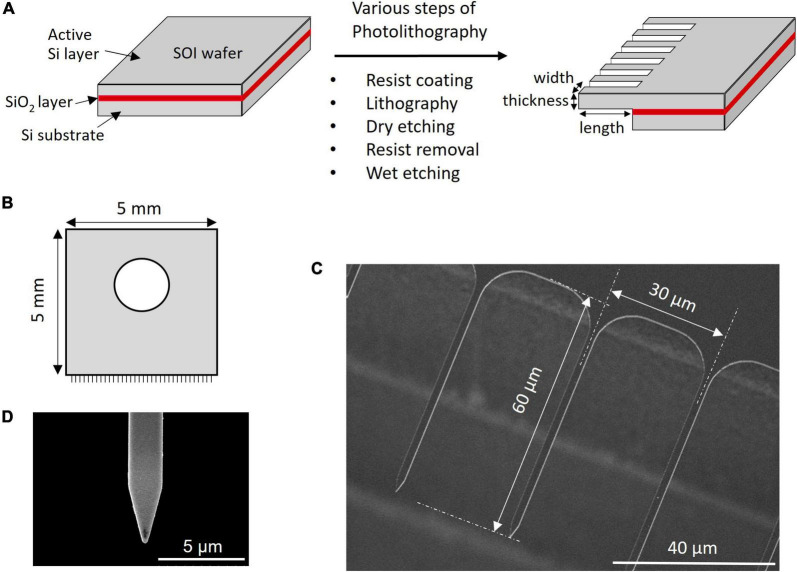
Microneedle array preparation and SEM image. **(A)** Steps involved in the fabrication of MNA. **(B)** Schematics of the MNA. SEM image of **(C)** array and **(D)** single needle.

Microneedle array chips with dimensions of 5 × 5 mm were fabricated on a silicon-on-insulator (SOI) wafer of 100 or 200 mm, a method commonly used in the micro-electro-mechanical systems (MEMS) fabrication process (Yamashita et al., 2018; Takeshita et al., 2020). The prismatic needles were fabricated horizontally from the substrate surface, resulting in a flat-comb-like appearance. The SOI wafer had a box layer of SiO_2_ between a thin active Si layer with a thickness of 2 μm and Si-substrate (Figure 1A). The SOI wafer was coated with photoresist and photolithographed using an MNA photomask to create the required comb-like pattern and dry etching was used to remove the silicon from the non-patterned area. The wafer was etched from the top and bottom sides and etching stopped when the SiO_2_ layer was reached. The MNA was then completed by removing the photoresist and removing the SiO_2_ layer by wet etching. The photomask was adjusted to produce MNA chips with needles of varying widths and lengths on a single wafer and the thickness was determined by the initial thickness of the active Si layer. MNAs with different needle dimensions were fabricated and tested for their efficiency in insertion into the target without the needles buckling or breaking.

### Microneedle Array Operating Setup for Plant Manipulation

This study developed the software and instrument set up for the semi-automatic insertion of MNA into the leaves and SAM (Supplementary Figures 1A,B). The setup consisted of a rectangular sample stage that was fixed and an MNA stage that could be moved horizontally to reach the sample stage for insertion. The MNA could be easily attached to or removed from the stage using a screw that fits a hole in the center of the MNA chip. The sample stage could be moved in the Y direction and tilted with a tilt stage. The MNA stage could be moved in the X and Z directions using micrometers attached to the setup. The MNA stage was also connected to a piezoelectric element that can oscillate in the direction of the needle axis with a frequency of ≤5 kHz. Specially developed software was used to control the movement and oscillation of the MNA stage. Parameters such as speed and oscillation conditions were input into the software and the insertion step automatically went through the steps of approach, dwell, and retraction of the MNA stage.

### Buckling Load Measurement of Microneedle Array

The buckling load of the MNA was measured using a micro-force sensor FS1M-0.1NP (THK Precision Co., Ltd., Tokyo, Japan). The pedestal part containing the sample holder of the plant manipulator was removed and a micro-force sensor was mounted (Supplementary Figure 2). The position was adjusted so that the tip of the MNA was in contact with the sensing probe part of the micro-force sensor. While observing using a stereomicroscope, the needle was pushed toward the probe until it buckled. The data from the micro-force sensor were processed using PicoScope^®^ PC Oscilloscope (Pico Technology, Saint Neots, United Kingdom) and PicoScope 6 software.

### Plant Materials and Growth Conditions

*Arabidopsis thaliana* ecotype Colombia-0 seeds were sterilized with 10% bleach for 20 min, washed with sterile water, and then planted on half-strength Murashige and Skoog (MS) medium containing 2% glucose, 0.1% MS vitamins, 0.06% MES [2-(*N*-morpholino)ethanesulfonic acid], 0.1% plant preservative mixture (Nacalai Tesque, Kyoto, Japan), and 0.25% Phytagel™ (Sigma–Aldrich, MO, United States) at pH 5.8. The plates were kept at 4°C for 48 h and then transferred to a plant incubator at 22.5°C and 16/8-h light/dark cycle. After approximately 8–10 days, *A. thaliana* seedlings were transferred to soil for further growth at 22.5°C, 16/8-h light/dark cycle.

Cre-responsive xGxGUS reporter plants of *A. thaliana* were obtained using the plasmid pCAMBIA-N-xGxGUS, as described below, where the reporter system had a GFP fragment with a transcription termination signal flanked between two *loxP* sites, followed by a sequence encoding GUS. *A. thaliana* (Col-0 strain; Inplanta Innovations, Inc., Yokohama, Japan) plants were cultivated at 23°C under constant light. To obtain Cre-responsive xGxGUS reporter plants of *A. thaliana*, a flower bud was dipped in a culture of *Agrobacterium* cells carrying pCAMBIA-N-xGxGUS (Furuhata et al., 2019) containing 5% w/v sucrose and 0.025% Silwet L-77 (Biomedical Science, Tokyo, Japan) for 3 min. After inoculation, the plants were placed under a cover to maintain high humidity and cultivated overnight under relatively weak light conditions. Subsequently, the plants were cultivated under normal light conditions until seed formation. Seeds were collected and sown on a GeM agar plate (0.5% agar, 20 g/L glucose, 0.6 g/L MES, 1× Gamborg’s B5 Salt Mixture, 1× MS vitamins, pH 5.7 adjusted with KOH) supplemented with 200 μg/mL cefotaxime and 20 μg/mL hygromycin. After germination of the rhizome, plants (T_1_) emitting green fluorescence were transferred to the soil (vermiculite). T_2_ generation plants cultivated from T_1_ seeds were used in our experiments.

*Glycine max* seeds from the cultivar ‘Tsurunoko-daizu’ (Takii Seed Co., Ltd., Kyoto, Japan) were sterilized by gas sterilization using bleach and hydrochloric acid at a ratio of 25:1. The sterilized seeds were kept in a desiccated chamber until further use. The seeds were rinsed with distilled water and germinated overnight on moist filter paper in an incubator set to 30/25°C in a 16 h/8 h light/dark cycle and 70–80% humidity. The next day, the cotyledon was removed and the embryonic axis containing SAM was excised while observing under a stereomicroscope (SZX10, Olympus, Tokyo, Japan). The leaf primordia were removed to expose the SAM and the explants were kept in sterile water until the Cas9 RNP delivery experiments.

### Elastic Modulus Measurements of Leaf Tissues and Shoot Apical Meristem

**FIGURE 2:**
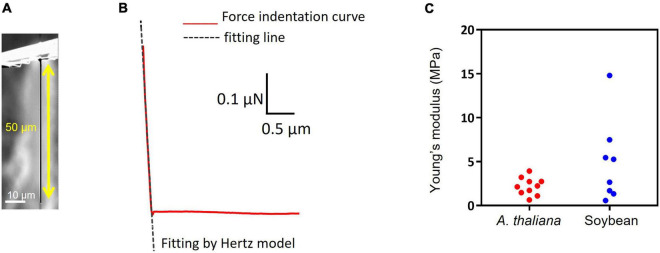
Stiffness measurement and insertion efficiency result. **(A)** Image of pillar indenter. **(B)** An example force-indentation curve of pillar indenter. The dashed line shows fitting by the Hertzian model. **(C)** Young’s modulus results of *Arabidopsis thaliana* leaf and soybean SAM.

The stiffness of *A. thaliana* leaves and SAMs was evaluated using an atomic force microscope (AFM, CellHesion^®^ 200, JPK BioAFM, Bruker, Berlin, Germany) at a setpoint of 1000 nN and an approach speed of 5 μm/s using the cantilever SD-PXL-NCL (NanoWorld AG, Neuchatel, Switzerland). For the measurements, the SD-PXL-NCL cantilever was modified into a cylindrical shape with a diameter of 1 μm and a length of approximately 50 μm (Figure 2A) using a focused ion beam (FIB) instrument (SMI500, Hitachi High-Tech Science, Tokyo, Japan). Hertz model was employed for the calculations. Hertz model describes the indentation of homogeneous elastic material by a stiff material with a defined geometry. Although cells are viscoelastic and non-homogeneous bodies and do not meet these requirements, many studies have been reported on the application of the Hertz model for biological samples because the Hertzian contact mechanics holds correct for a small indentation depth and a small indenter compared to the size of the sample (Kontomaris and Stylianou, 2017; Akita et al., 2020; Kontomaris and Malamou, 2020).

In this study, a pillar indenter is used and the corresponding Hertzian contact mechanics can be described as a linear curve, the equation is as follows:

$$F=\frac{2\,r\,E}{\left(1-v^{2}\right)}\,d$$

where *F* is the applied force, *E* is the Young’s modulus, *v* is the Poisson’s ratio (0.5), *d* is the indentation depth, and *r* is the tip radius (0.5 μm). Figure 2B shows a force-indentation curve obtained by pillar indenter and the dashed line shows fitting by Hertz model using a least-squares fit with the Levenberg-Marquardt algorithm (JPK DP, Data Processing Software Manual, Version 6.0, 11/2018). Young’s modulus *E* can be calculated from the slope of the fitted line for the force curve because the Poisson’s ratio and the tip radius were defined.

### Expression and Extraction of Cre Recombinase, Cas9, and Cas9-Green Fluorescent Protein

Cre proteins were expressed using *Escherichia coli* strain BL21(DE3) following the procedure described in a previous study (Furuhata et al., 2019). Nuclear localization signal (NLS)-fused Cas9 derived from *Streptococcus pyogenes* was expressed using the pET-28b-Cas9-His plasmid vector (#47327, Addgene, United States) following the same procedure as described in a previous study (Yamagishi et al., 2019). For Cas9-GFP, *E. coli* strain BL21(DE3) was transformed with the expression vector pET-Cas9-GFP-NLS-His, generated from pET-28b-Cas9-His (Supplementary Figure 3). *Escherichia coli* cells were cultured at 37°C for 3 h with shaking. Protein expression was induced using 0.1 mM isopropyl β-D-1-thiogalactopyranoside (IPTG). After 2.5 h, the collected cells were lysed with lysis buffer (50 mM Tris–HCl, 500 mM NaCl, 10% glycerol, 10 mM imidazole, 1 mM phenylmethylsulfonyl fluoride, 1 mM DTT, pH 8.0). Cas9 protein was purified using Ni-NTA agarose (Qiagen, Hilden, Germany) with wash buffer (50 mM Tris–HCl, 500 mM NaCl, 10% glycerol, 20 mM imidazole, pH 8.0) and eluted with elution buffer (50 mM Tris–HCl, 500 mM NaCl, 10% glycerol, 500 mM imidazole, pH 8.0). The eluate was further purified by gel filtration (HiPrep 16/60 Sephacryl S-200 HR; GE Healthcare, IL, United States) using buffer A [20 mM HEPES, 500 mM NaCl, 10% glycerol, and 1 mM dithiothreitol (pH 7.4)] to completely remove imidazole and stored at −80°C until further use.

### Cre Recombinase Delivery and β-Glucuronidase Staining

New MNAs were washed with ultrapure water (Kanto Chemicals Co., Inc., Tokyo, Japan) and treated with a UV-ozone cleaner (UV253, Filgen Inc., Nagoya, Japan) for 15 min. If reusing the MNA, it was initially treated with piranha solution (sulfuric acid and hydrogen peroxide in a 4:1 ratio) at 70°C for 30 min to remove the organic matter from the surface, followed by UV-ozone surface cleaning for 30 min. Cre recombinase (10 μM) was dissolved in sterile distilled water with an adjusted pH of 5.8 and 0.05% of Silwet L-77. The cleaned MNA was placed on the array stage and a leaf from the xGxGUS *A. thaliana* reporter plant was kept in place on the sample stage using adhesive double tape (NWR15, NicetackTM, Nichiban Co., Ltd., Tokyo, Japan). The array stage was moved toward the sample stage so that when observed through the stereomicroscope, the space between the needle tip and leaf was approximately 20–30 μm. Around 10 μL of 10 μM Cre solution containing 0.05% silwet was added to the space between the MNA and leaf tissue and automatic insertion was started (Supplementary Figure 4A). Throughout the experiment, the incubation time was maintained at 60 s and the approach and retraction speed were set to 10 μm/s. After the specified time, the MNA was automatically retracted from the sample. The leaf samples were carefully removed from the stage and incubated on an MS media plate for 40 h.

To visualize GUS expression, the leaf samples were incubated in 0.5 mg/mL of X-Glucuronide (X-Gluc, Carbosynth, Compton, United Kingdom) dissolved in staining buffer [20% (w/v) methanol, 0.1% Triton X-100, 50 mM NaH_2_PO_4_, pH 7.0] for 5 h at 37°C. The leaves were then transferred to a decolorizing solution of ethanol and acetic acid in a ratio of 6:1 and kept overnight for decoloration and the solution was exchanged with 99.5% ethanol as appropriate. When the leaves turned white, the leaves were washed with sterile distilled water, kept on glass slides, and observed blue spots. Images were captured using an inverted microscope (IX71, Olympus). For observation of Cas9-GFP/sgRNA delivered into *A. thaliana* leaves, a confocal laser microscope (A1, Nikon, Tokyo, Japan) was used. Nuclear staining was performed for leaves after MNA insertion by soaking in 10 μg/ml DAPI solution and incubating for 1 h at room temperature.

### Synthesis of sgRNA Targeting the Soybean’s PDS11/18 Gene

The gene for carotenoid biosynthesis, PDS11/18, was the target gene of interest in soybean (Du et al., 2016). Synthesis of sgRNA was carried out using the method described in a previous study (Yamagishi et al., 2019). sgRNA containing a poly U sequence was used because of its increased cleavage efficiency. The Forward oligo, which has a T7 promoter sequence, was mixed with the Reverse oligo, which has a polyadenine sequence. The two oligo DNAs were annealed and polymerized in a 5′ to 3′ direction by the Klenow fragment. Next, the sgRNA was transcribed using the synthesized DNA fragment and RiboMax™ Large Scale RNA Production Systems (Promega, WI, United States). Transcribed sgRNA was isolated using ISOGEN II (Nippon Gene Co., Ltd., Tokyo, Japan). Sequence information is provided in Supplementary Table 1.

### *In vitro* Cleavage Activity of Cas9 RNP

Genomic DNA was extracted from soybean SAM using the ISOSPIN Plant DNA kit (Nippon Gene Co., Ltd.) and PCR was performed using primers specific for the sgRNA cleavage site. Then, 5 pmol Cas9 and 15 pmol sgRNA were prepared and mixed with the PCR-amplified PDS11 gene and incubated at 37°C for 1 h. The cleaved DNA fragments were confirmed by electrophoresis on a 2% TBE gel.

### Delivering Cas9 Ribonucleoprotein Into Soybean’s Shoot Apical Meristem and Next-Generation Sequencing

A 10 μM Cas9 RNP mixture in nuclease-free water containing 5 mM MgCl_2_, 0.4 U/μL RNase inhibitor (Nippon Gene Co., Ltd.), 10.1 μM of sgRNA, 0.05% Silwet, and 10 μM Cas9 was prepared. The mixture was mixed by tapping and incubated on ice for 15 min. The parameters for the instrumental setup to deliver the Cas9 RNP into the SAM of soybean using MNA were similar to those for Cre protein delivery into *A. thaliana* leaves (Supplementary Figure 4B). The soybean embryonic axis was placed horizontally and attached to the sample stage using a cover glass and secured using a small magnet on the top. The SAM part of the axis faced the array stage on the other side. For the delivery, the SAM containing the sample stage was brought to within approximately 10 μm of the MNA stage. Twenty microliters of 10 μM Cas9 RNP solution was dropped between them and automatic insertion was started at a speed of 10 μm/s. After the first insertion, the above steps were repeated 5 times with the MNA position changing each time, either 20 μm vertically or 15 μm horizontally. To prevent the sample from drying, 5 μL of 10 μM Cas9 RNP solution was added after each insertion. After Cas9 RNP was transferred into the SAM, the explants were placed in an upright position on a medium plate containing 0.23% MS inorganic salt, 0.1% MS vitamin solution, 3% sucrose, 0.1% plant preservative mixture, 0.01% myo-inositol, and 0.25% Phytagel™ at pH 5.8. After 16 h of incubation at 30°C and 70–80% humidity with light, the SAM was excised and used for PCR.

For next-generation sequencing (NGS) sample preparation, the MNA-inserted part of the SAM was cut out after 16 h of incubation and genomic DNA was extracted using the ISOSPIN Plant DNA kit (Nippon Gene Co., Ltd.). We conducted PCR to amplify the target sequence using genomic DNA as a template for NGS. Detailed information regarding the sequences used for NGS is provided in Supplementary Table 2 and Supplementary Figure 5. After PCR amplification, the DNA was purified using the Wizard SV Gel and PCR Clean-Up System (Promega, United States). Bioengineering Lab (Kanagawa, Japan) completed NGS analysis. Briefly, PCR amplicons were purified using AMPure XP (Beckman Coulter, CA, United States). For the preparation of the pooled sequencing libraries, a second PCR was performed using the indexing primers. After purification, the libraries were sequenced using MiSeq system and MiSeq Reagent Kit v3 (Illumina, CA, United States). The obtained data were analyzed using CRISPResso2 to confirm that the mutation occurred in the Cas9 cleavage site.

## Results

### Dimensions of Microneedle Array Required for the Successful Insertion Into Plant Tissues

Considering the approach to the soybean SAM with a height of approximately 30 μm (Yoshikawa et al., 2013), we design a novel MNA, where needles over 30 μm in length are aligned sidewise to the silicon wafer, giving it a comb-like appearance (Figures 1B,C). The microneedles tested in this study were uniformly 2 μm in thickness and 30 μm in pitch, with widths of 1 or 2 μm and lengths of 40, 60, or 100 μm. As shown in Figure 1C, a round structure with a radius of 10 μm was designed at the root to reduce the load concentrated at the root of the needle. The difference in a wedge-shaped tip or rectangular tip of 1 μm width and 60 μm length when inserted into *A. thaliana* leaf tissue is shown in Supplementary Video 1. A graphical representation of a rectangle tip and wedge-shaped tip is shown in Supplementary Figure 6. The rectangle tip means that the shape of a single needle is a rectangle. The wedge-shaped means the tip is formed in a tapering shape. The vertex angle of the triangle of the planar part that makes up this wedge is 30° (Figure 1D). The buckling during insertion and the number of broken needles indicated that the wedge-shaped tip structure had better insertion. Thus, for further experiments, we used MNA-containing needles with wedge-shaped tips. Needle pitches of 10 and 60 μm were also tested (results not shown). The 10 μm resulted in the entire tissue being indented and the needle did not penetrate. Although the MNA with 60 μm pitches successfully penetrated the leaf tissue, the total number of needles in an MNA is less thus the closely spaced 30 μm was adopted. An MNA chip with a pitch of 30 μm contained 167 microneedles.

The Young’s modulus of mammalian cells is usually in kilopascal (kPa) (Kagiwada et al., 2010), while the values of a plant cell are in megapascals (MPa) due to the cell wall. The Young’s modulus measurements of *A. thaliana* leaf and soybean SAM showed comparable results (leaf: 2.2 ± 1.0 MPa and SAM: 4.9 ± 4.7 MPa; Figure 2C), suggesting that the parameters of the needle, such as length and width for successful insertion, were common to both tissues. If the insertion fails, the needle buckles and eventually breaks. Buckling occurs when the load exceeds the strength of the needle, which is a parameter that must be considered for successful penetration into tissues. The buckling load was measured using a micro-force sensor. A higher buckling load means the needle is less likely to break. In our experiments, needles with widths of 2 μm and lengths of 40 or 60 μm showed high buckling loads (Supplementary Table 3). Needles with lengths of 100 μm and widths of 1 or 2 μm showed low buckling loads. Supplementary Video 2 shows a needle of 1 μm width and 100 μm length buckling when MNA was inserted into *A. thaliana* leaves. In contrast, needles with widths of 1 and 2 μm and lengths of 40 and 60 μm were inserted successfully (Table 1 and Supplementary Video 3). The insertion efficiency was calculated from the number of unbroken needles in an MNA after insertion to the total number of needles in that before insertion. The insertion efficiency was highest with a width of 2 μm and length of 60 μm in both *A. thaliana* leaf and soybean SAM (Table 1). While considering the insertion depth needed for SAM tissues, MNA with 2 μm width, 30 μm pitch, and 60 μm length (MNA236), with a buckling load of 0.68 mN, were found to be effective for insertion into *A. thaliana* leaf and soybean SAM.

**TABLE 1:** Insertion efficiency results on *Arabidopsis thaliana* leaf tissue and *Glycine max* SAM.

Length (μm)	40		60	
Width (μm)	*A. thaliana*	*G. max*	*A. thaliana*	*G. max*
1	77.4%	77.9%	50.4%	60.2%
2	96.3%	97.2%	97.2%	98.0%

**FIGURE 3:**
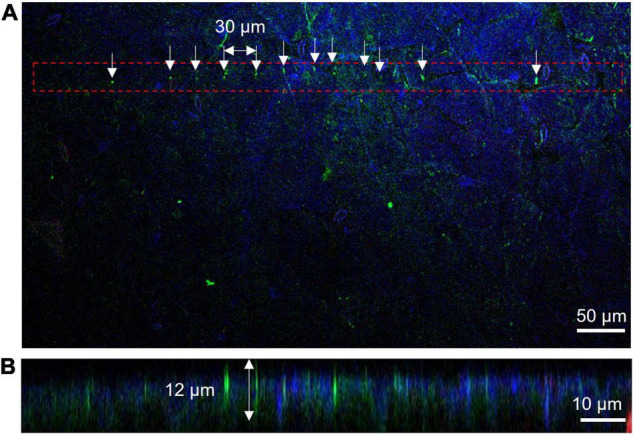
Measurement of insertion depth by fluorescent observations of Cas9-GFP (green) and nucleus stained with DAPI (blue). **(A)** View from the top side of the leaf. The MNA contacted area was marked with the red dash line and the needle insertion points were marked by arrows. **(B)** Side view of leaf cross-section. The slice was obtained along with the aligned green dots in Panel **(A)**.

Insertion depth for MNA236 was measured by fluorescent observations using Cas9-GFP/sgRNA immobilized arrays. After MNA insertion into *A. thaliana* leaves, DAPI-stained leaves were observed under a confocal microscope (Figures 3A,B). Figure 3A is the view from the top side of the leaf where the MNA contacted area was marked with the red dash line and the needle insertion points were marked by arrows. Figure 3B is the side view of the leaf cross-section. GFP fluorescence was observed at the insertion points with 30 μm spacing and a depth of approximately 12 μm. The DAPI-stained nuclei (blue) cannot be observed deeper than 12 μm because the excitation laser beam could not reach further than this. In other words, the Cas9-GFP protein was delivered and reached the depth limit where it could be detected by ordinary confocal microscopy. In this experiment, the surface of the MNA236 was cleaned with no special treatment. This result shows that physisorbed Cas9-GFP can reach deep into the leaf tissue.

### Cre Recombinase Delivery Into Reporter Plants of *Arabidopsis thaliana*

**FIGURE 4:**
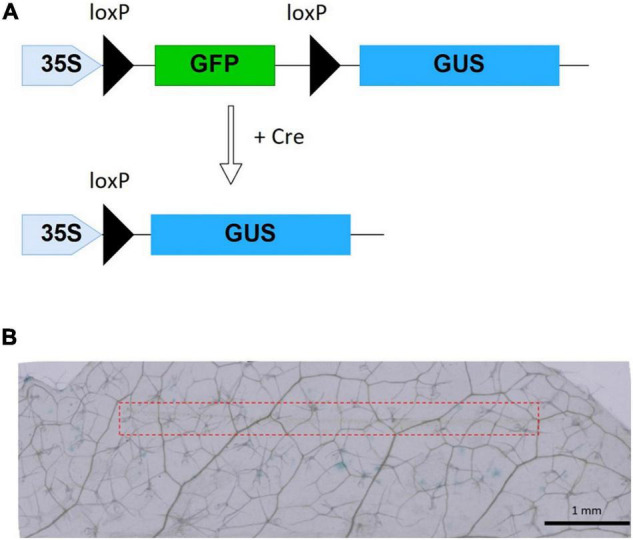
Cre recombinase reporter system and Cre delivery results. **(A)** xGxGUS reporter system of *A. thaliana.* GFP, green fluorescent protein; GUS, β-glucuronidase; *loxP*, Cre recombinase target sequence. **(B)** GUS staining results after 40 h incubation with 10 μM Cre insertion using MNA, the red dash line represents the area of MNA insertion.

Successfully delivered Cre recombinase by MNA into reporter plants excises the sequence encoding GFP flanked between two *loxP* sites, resulting in the expression of the GUS gene (Figure 4A). As negative control experiments, we performed staining treatment for leaves without any insertion, leaves with insertion where protein solution was not used, and leaves inserted with MNA followed by dropping of protein solution. The control tests showed no blue spots representing GUS expression. Whereas, all of the leaves with Cre-delivered by MNA insertion showed blue spots (Figure 4B), meaning that the efficiency of Cre recombination per trial was 100%. On the 23 leaves used for Cre delivery, 271 blue spots were observed, giving an average of 12 blue spots per leaf. Additionally, there was no visible damage to the leaves after MNA insertion and their green color were retained even after incubation for 40 h (Supplementary Figure 7). The oscillation of MNA was investigated in the Cre delivery experiment, but it had no significant effect on the delivery efficiency (data not shown).

### Cas9 Ribonucleoprotein Delivery Into Shoot Apical Meristem of Soybean and Next-Generation Sequencing Analysis

A PCR product of the PDS11 gene of 380 bp was subjected to an *in vitro* cleavage assay and its cutting site with Cas9 RNP was almost at the mid-position in the fragment, which generated 185 bp and 195 bp fragments. Cut bands overlapped and were observed as a single band, confirming the activity of purified Cas9 RNP (Supplementary Figure 8).

**FIGURE 5:**
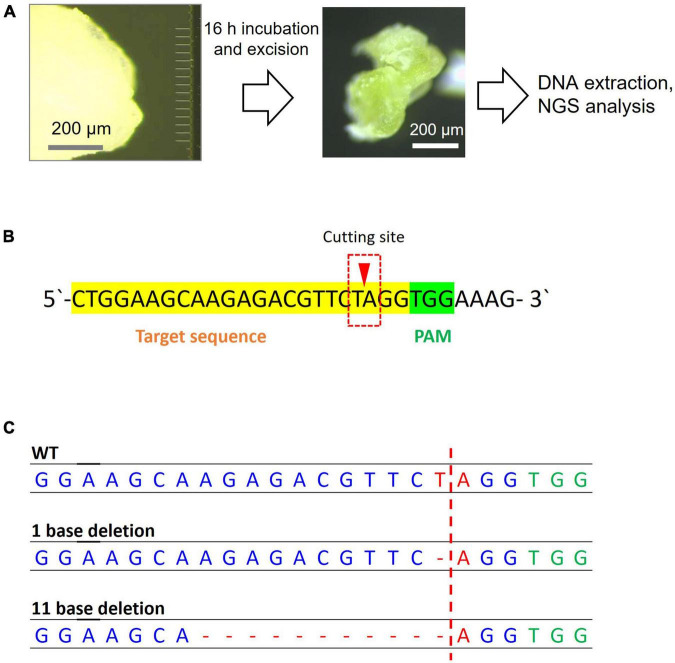
Next-generation sequencing sample preparation and analysis results for genome editing of soybean SAM. **(A)** Steps of MNA insertion into soybean SAM and sample preparation for NGS. **(B)** The sgRNA target sequence of the PDS11 gene. The reads that have a mutation in the red box were counted. **(C)** The mutation patterns were found in the target sequence by NGS analysis.

After Cas9 RNP delivery, samples were incubated for 16 h at 37°C, and the inserted portion was cut out for genomic DNA extraction (Figure 5A) and NGS analysis. The excised SAM showed a greening effect, indicating that the viability of tissues was retained (Figure 5A). Among the obtained read sequences, the indels identified near the sgRNA cleavage site (Figure 5B), three or four bases upstream of the PAM sequence, were counted and their percentages were calculated. Among the total reads of approximately 3 × 10^4^, an 11 bp deletion with 7 reads and 1 bp deletion with 3 reads were observed in the Cas9 RNP complex-delivered samples (Figure 5C). We believe that the 11 bp deletion was caused by the cutting of PDS11 gene with Cas9 RNP delivered by MNA and subsequent non-homologous end-joining although the 1 bp deletion could be occurred by an error of PCR or NGS analysis. A fewer number of edited reads containing deletion were probably due to a large number of wild-type cells in the sample cut out. Since it is likely that only a few cells in the SAM were genome-edited, some reads containing deletions could not be amplified. The mutation rate calculated from the result was 0.03%. The volume of excised tissue was approximately 10^7^ mm^3^. Assuming that the size of cells in the soybean tissue was 10^3^ μm^3^ (Laufs et al., 1998), 2 × 10^4^ cells were present, and if we estimated the number of cells that each needle was able to insert to be 100, we needed to evaluate the value of efficiency 200 times higher. Therefore, large-scale deletions occurred at a frequency of approximately 6% in the cells where the needle was inserted. The growing ability of SAM was not hampered by MNA insertion as shoots or roots started to develop when incubated for over 2 days (Supplementary Figure 9).

## Discussion

In Japan, genome-edited plants produced from molecular breeding based on gene disruption are not considered genetically modified organisms because they are indistinguishable from naturally occurring plants. When genome editing is performed using plasmid DNAs or DNA-based vectors, it is necessary to remove any transgenes that remain in the genome DNA by backcrossing, which is a time-consuming process. In contrast, protein-based genome editing does not have this problem. In this study, SAM was modified by an *in planta* method with direct protein delivery. This method allowed the time-consuming tissue culture step to be skipped, which is a huge advantage for editing plants that are difficult to regenerate from protoplasts.

DNA-free gene modification in plant cells with an intact cell wall was achieved by the direct delivery of plant genome editing systems using MNA. By designing the MNA with microneedles of an appropriate size, we achieved transfer without losing viability. The MNA transferred the genome editing systems into the mesophyll layers of plant cells, bypassing the epidermal cells, which are often considered the toughest barrier in a plant cell. In the case of SAMs, the whole plant can be directly grown by incubating the axis of the modified SAM into the culture medium, skipping the time-consuming tissue culture step. Another notable feature of this method is that we can easily customize the needle dimensions of the MNA based on the stiffness of the target tissues.

The buckling properties of the MNA with lengths of 40, 60, and 100 μm and widths of 1 and 2 μm were compared. As shown in Supplementary Table 3, even though the 2 μm width and 40 μm length had the highest buckling load, longer needles that could reach the subepidermal L2 layer of the target soybean SAM were necessary. Thus, considering the GUS staining results and buckling load, MNA 236 (2 μm width, 30 μm pitch, 60 μm length) was used for further experiments.

We obtained a GUS expression efficiency of 100% per delivery with Cre recombinase incorporation into reporter plants of *A. thaliana* by MNA. Negative control leaves of Cre recombinase delivery without MNA insertion did not show any GUS staining blue spots, indicating that MNA facilitates the transport of Cre recombinase deep inside the leaf cells by breaking through the intact cell wall. As shown in Figure 4B, GUS-expressed tissues were observed near the inserted areas as well as in the cells away from the insertion point. This can be attributed to the movement of the GUS protein fused with an extracellular secretory signal from the inserted point to other areas through the leaf veins during the incubation period. It is also possible that Cre protein released from MNA can be transported into non-adjacent cells or GUS-expressing cells from wound tissue and can travel *via* intercellular movement (Hertle et al., 2021).

In this study, we targeted PDS11/18, a gene known to be related to pigment synthesis in soybean (Du et al., 2016). The primers for amplification used in the NGS analysis were designed based on PDS11 and contained three-base mismatches for PDS18 in the forward and reverse primers, and the sequences of the amplicons obtained for this purpose were all derived from PDS11. Although the mutation in PDS18 was not analyzed, it was assumed that a similar deletion would be found. An estimated genome editing efficiency for SAM was 6%. While considering some of the successful results from the latest research on genome editing in SAM, the percentage of samples that carried the mutant alleles in tissues at the initial stage of our research was high. For example, Liu et al. (2021) used *in planta* particle bombardment-mediated delivery of CRISPR/Cas9 in wheat embryos and 6.9% of the targeted embryos carried the mutant alleles in tissues. We expect to achieve comparable efficiency using MNA. On the other hand, other methods cannot control the target point in the tissue, which in turn requires the time-consuming process of selecting the successfully delivered samples for further analysis. As mentioned in the results, the retention of green color in *A. thaliana* leaves and shoot development in soybean SAM during incubation confirms the viability of tissues following MNA insertion.

In conclusion, we demonstrated an efficient method for the direct delivery of genome editing materials, Cre recombinase and Cas9 RNP, into plants using MNA. We successfully transferred Cre recombinase into the model plant *A. thaliana* leaf tissue and Cas9 RNP into the SAM of a practical plant soybean without any visible physical damage to the samples that could lead to tissue death.

## Data Availability Statement

The original contributions presented in this study are included in the article/Supplementary Material, further inquiries can be directed to the corresponding author.

## Author Contributions

MH and AV performed the experiments. CN conceived the idea and coordinated all tasks. AV, AY, and CN wrote the manuscript. YF and YK designed the Cre reporter system, synthesized the Cre and Cas9 proteins. NM, TT, and TK designed and fabricated MNA. FI created an MNA operating setup. MK and TY optimized the SAM isolation protocol. All authors contributed to the article and approved the submitted version.

## Conflict of Interest

The authors declare that the research was conducted in the absence of any commercial or financial relationships that could be construed as a potential conflict of interest.

## Publisher’s Note

All claims expressed in this article are solely those of the authors and do not necessarily represent those of their affiliated organizations, or those of the publisher, the editors and the reviewers. Any product that may be evaluated in this article, or claim that may be made by its manufacturer, is not guaranteed or endorsed by the publisher.
