# Supplementary material for: Microneedle Array-Assisted, Direct Delivery of Genome-Editing Proteins Into Plant Tissue
Source: Front Plant Sci. 2022 Jun 24;13:878059. doi: 10.3389/fpls.2022.878059 (PMC9263851; doi:10.3389/fpls.2022.878059)
Supplement: Supplementary file 4 [file Data_Sheet_1.pdf]

Supplementary Material

1 Supplementary Figures, Videos, and Tables

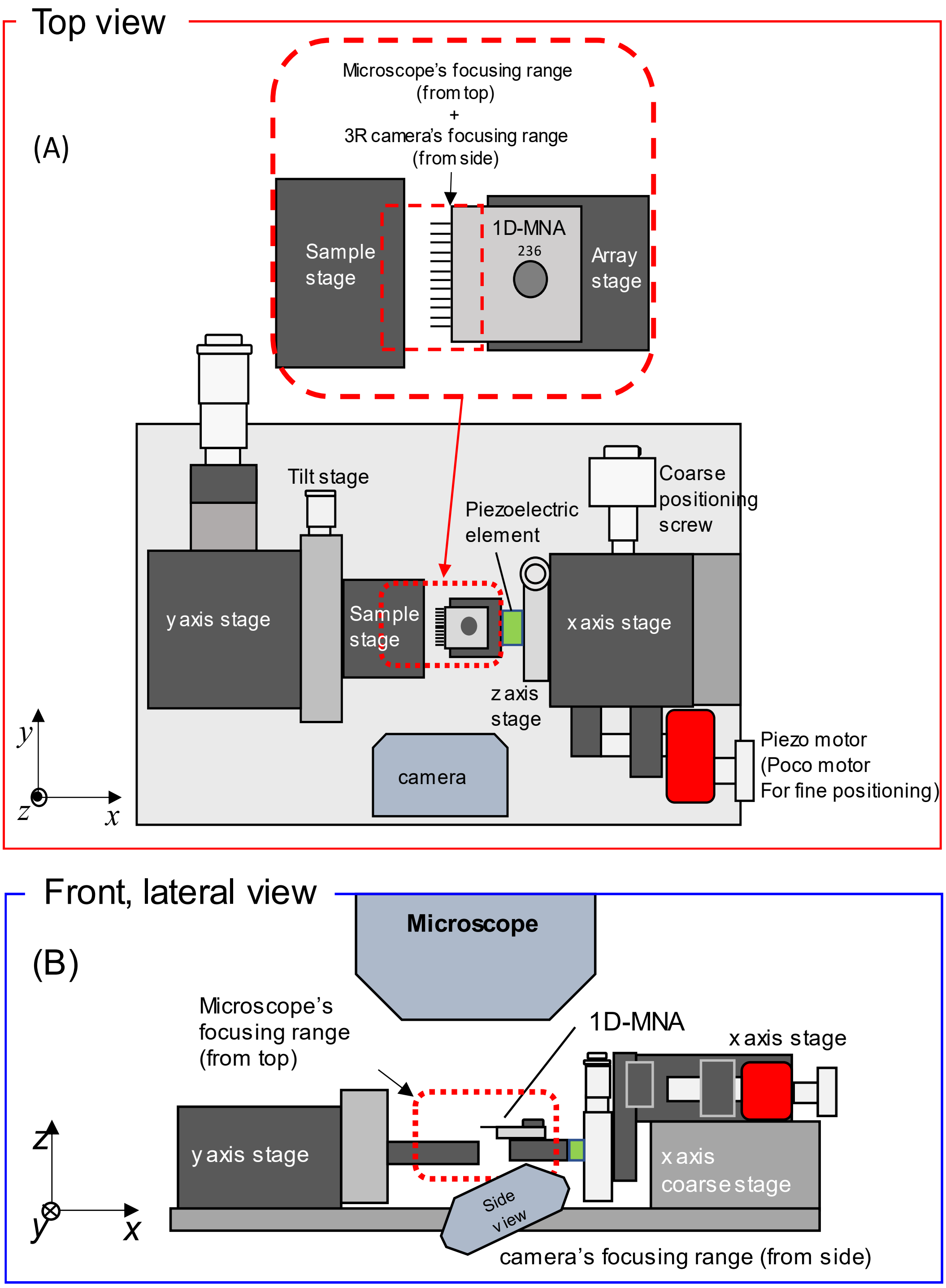

Supplementary Figure 1 Instrumental setup for MNA manipulation. (A) View from the top of the setup. (B) Front or lateral view of the setup.

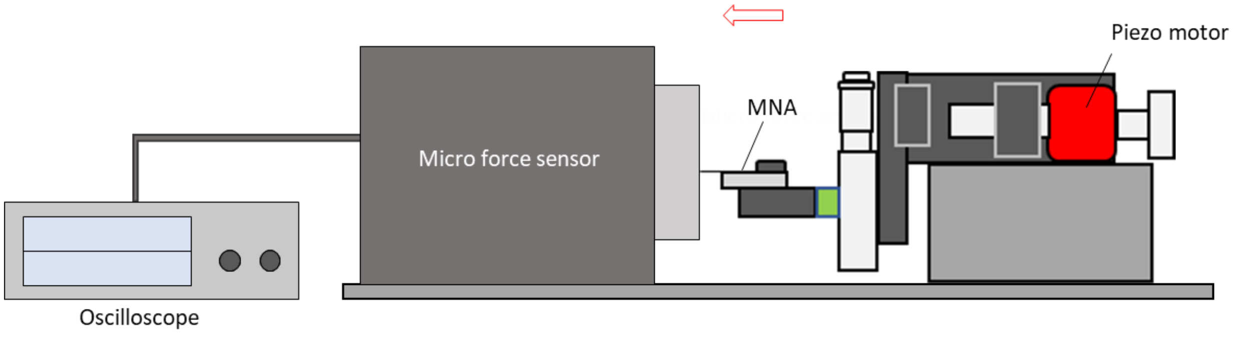

Supplementary Figure 2 Micro force sensor alignment for buckling measurements.

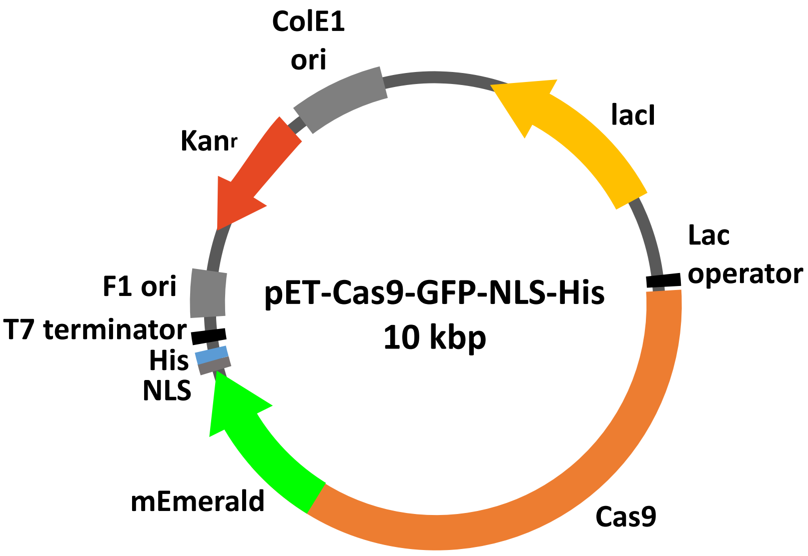

Supplementary Figure 3 Plasmid map of pET-Cas9-GFP-NLS-His.

(A)

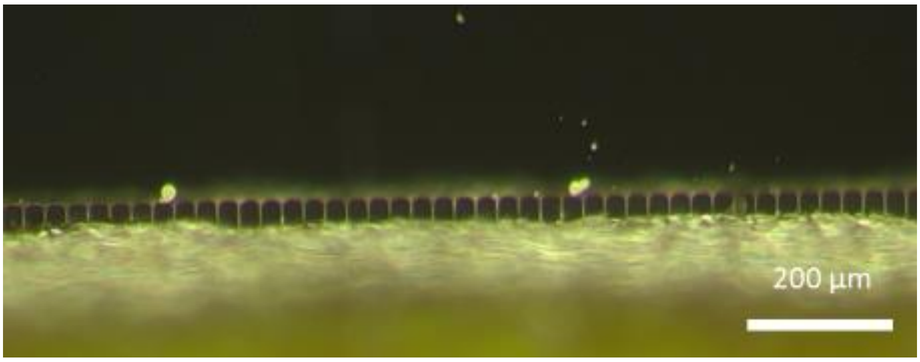

(B)

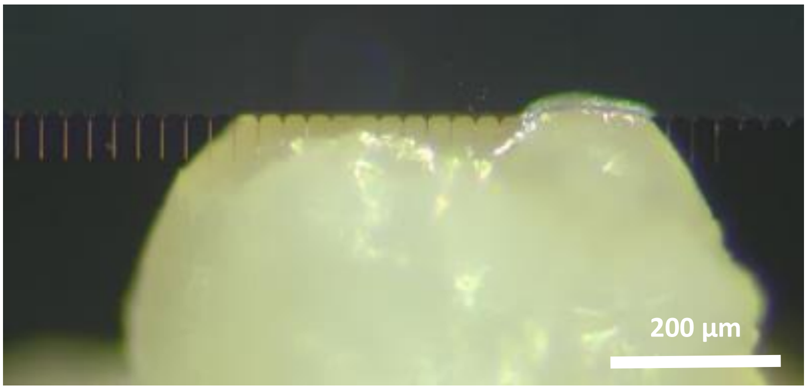

Supplementary Figure 4 Images of MNA236 insertion in (A) *A. thaliana* leaf and (B) soybean SAM.

frontiers

AAAGGTATCCTGATATCATGTTGGATATTTAAAGACAAAAAAATTTGTAAACTAATCTTTGCACATTAGT
TTTGGCCTCTCTGCCTATTGGATTGATGCATGTTTCATTATTGCAGGATTGGCTGGTTTATCAACTGCAA
AATATTTGGCTGATGCTGGGCATAAACCTATATTGCTGGAAGCAAGAGACGTTCTAGGTGGAAAGGTT
TTCCTGCTAATTTAATCTCTTACGTCAATTAGTTGTCACTTTGTGTGCATTCTGCTCATTATTTTAGCATGC
TGTTTAATGAAATAAGAAATTTGTTTGTGTTGTTCCGTCATAATCTATTTGATGTGTTGGCTTTATAACTT
CATCATCAGGCTTGTTGAAGATTACCGCA

PCR primers Target sequence PAM sequence

Supplementary Figure 5 Sequence near the sgRNA target of the soybean PDS11 gene.

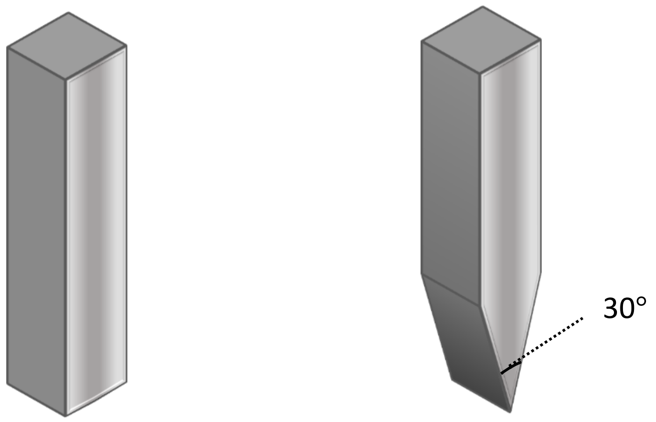

Supplementary Figure 6 Graphical representation of a needle in MNA with a rectangle tip (left) and wedge-shaped tip (right).

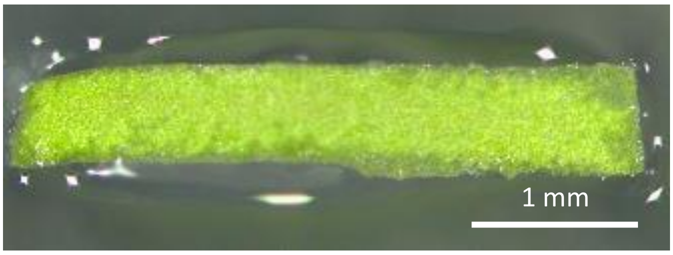

Supplementary Figure 7 MNA-assisted protein delivered *A. thaliana* leaf after 40 h incubation.

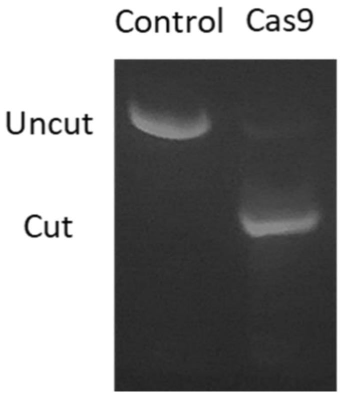

Supplementary Figure 7 Cleavage activity of Cas9/sgRNA *in vitro*.

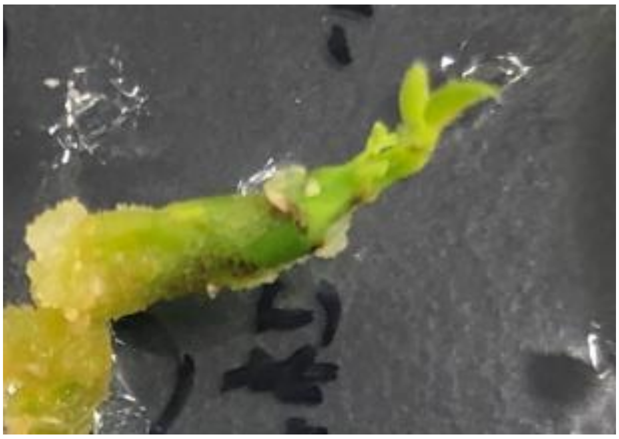

Supplementary Figure 8 MNA-assisted protein delivered soybean SAM after 2 days incubation.

Supplementary Video 1 The difference between needles of 1 µm width and 60 µm length MNA fabricated with wedge-shaped tip (left) and rectangular tip (right) when inserted into *A. thaliana* leaf tissues. A graphical representation of the needle shape is shown in the inset.

Supplementary Video 2 The buckling of a needle in the MNA with 100 µm in length and 1 µm in width when inserted into *A. thaliana* leaf.

Supplementary Video 3 Complete insertion of a needle in the MNA with 60 µm long needle with 2 µm width when inserted into *A. thaliana* leaf.

Supplementary Table 1 Oligo DNA sequences and synthesized sgRNA sequence.

Forward oligo	TAATACGACTCACTATAGGGAAGCAAGAGACGTTCTAGGGTTTTAGAG CTAGAAATAGCAAG
Reverse oligo	AAAAAAAGCACCGACTCGGTGCCACTTTTTCAAGTTGATAACGGACTA GCCTTATTTTAACTTGCTATTTCTAGCTCTAAAAC
Synthesized sgRNA sequence	GGAAGCAAGAGACGUUCUAGGGUUUUAGAGCUAGAAAUAGCAAGU UAAAAUAAGGCUAGUCCGUUAUCAACUUGAAAAAGUGGCACCGAG UCGGUGCUUUUUUU

Orange: T7 promoter sequence, Green: Target sequence for PDS11/18, Blue: Overlap sequence between Forward and Reverse oligos, Red: Seven U sequence

Supplementary Table 2 PCR primer used for Cas9 cleavage assay and NGS analysis.

PDS11 Forward	TGCGGTAATCTTCAACAAGCC
PDS11 Reverse	AAAGGTATCCTGATATCATGTTGGA
PDS11 Forward NGS	ACACTCTTTCCCTACACGACGCTCTTCCGATCTTGCGGTAATCTTCAAC AAGCC
PDS11 Reverse NGS	GTGACTGGAGTTCAGACGTGTGCTCTTCCGATCTAAAGGTATCCTGAT ATCATGTTGGA

Blue: Adaptor sequences used in TruSeq

Supplementary Table 3 Selected buckling loads for various microneedles.

Width (μm)	Length (μm)	Buckling load (mN)
1	40	0.36
	60	0.020
	100	0.030
2	40	1.4
	60	0.68
	100	0.17
